# Supplementary material for: Two original observations concerning bacterial infections in COVID-19 patients hospitalized in intensive care units during the first wave of the epidemic in France
Source: PLoS One. 2021 Apr 29;16(4):e0250728. doi: 10.1371/journal.pone.0250728 (PMC8084132; doi:10.1371/journal.pone.0250728)
Supplement: S1 Fig — (DOCX) [file pone.0250728.s001.docx]

S1 Fig. Persistence of bacteria in respiratory samples of COVID-19 patients with VAP.
