# Supplementary material for: Two original observations concerning bacterial infections in COVID-19 patients hospitalized in intensive care units during the first wave of the epidemic in France
Source: PLoS One. 2021 Apr 29;16(4):e0250728. doi: 10.1371/journal.pone.0250728 (PMC8084132; doi:10.1371/journal.pone.0250728)

S1 Table. Description of patients COVID-19 in ICU and comparison of patients with or without bacterial infections.


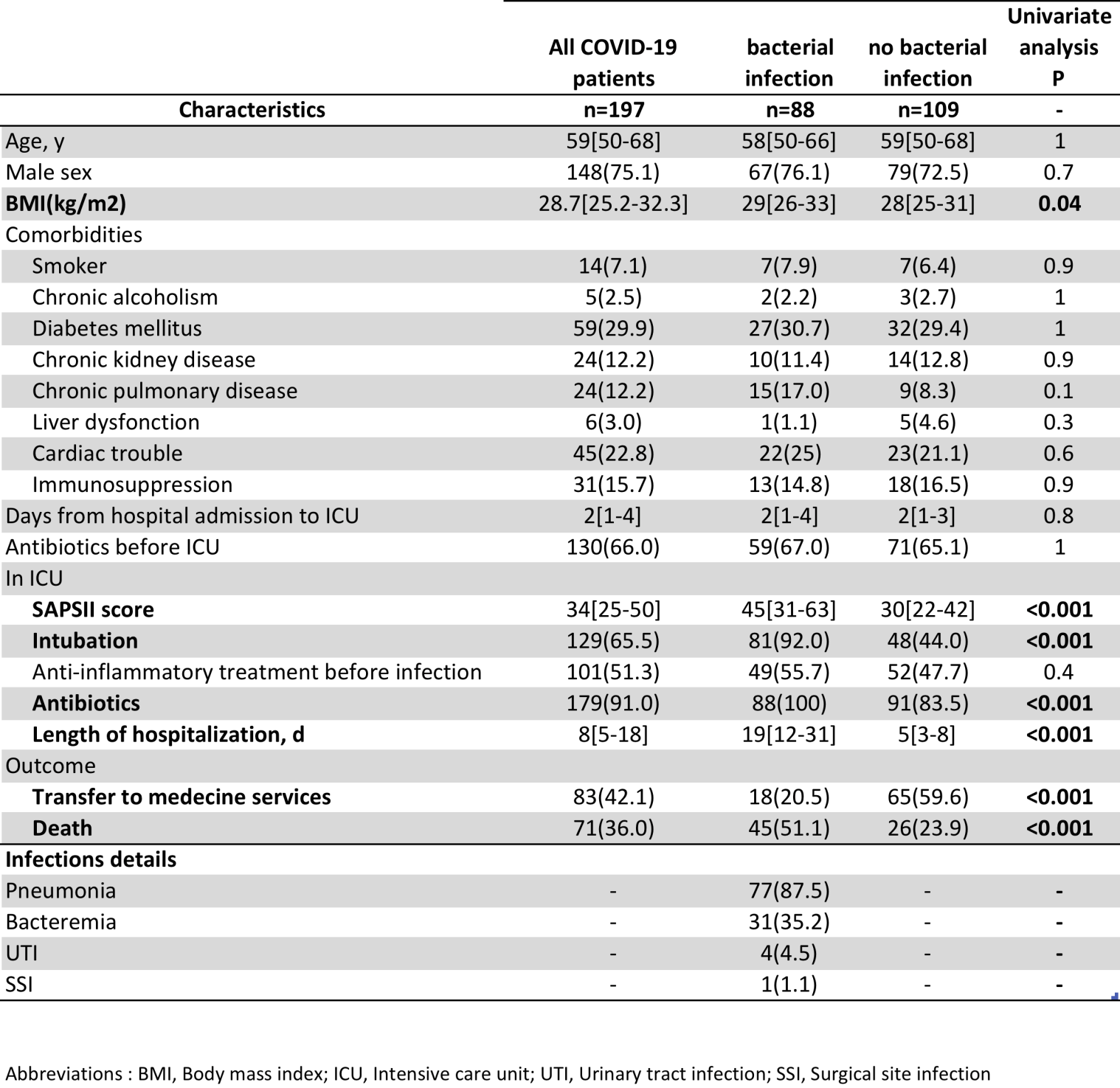

Supplement: S1 Table — (DOCX) [file pone.0250728.s002.docx]
