# Supplementary material for: Two original observations concerning bacterial infections in COVID-19 patients hospitalized in intensive care units during the first wave of the epidemic in France
Source: PLoS One. 2021 Apr 29;16(4):e0250728. doi: 10.1371/journal.pone.0250728 (PMC8084132; doi:10.1371/journal.pone.0250728)

S2 Table. Description of all bacteria responsible for ventilator associated pneumonia (VAP), bacteriemic VAP and primary bloodstream infection.


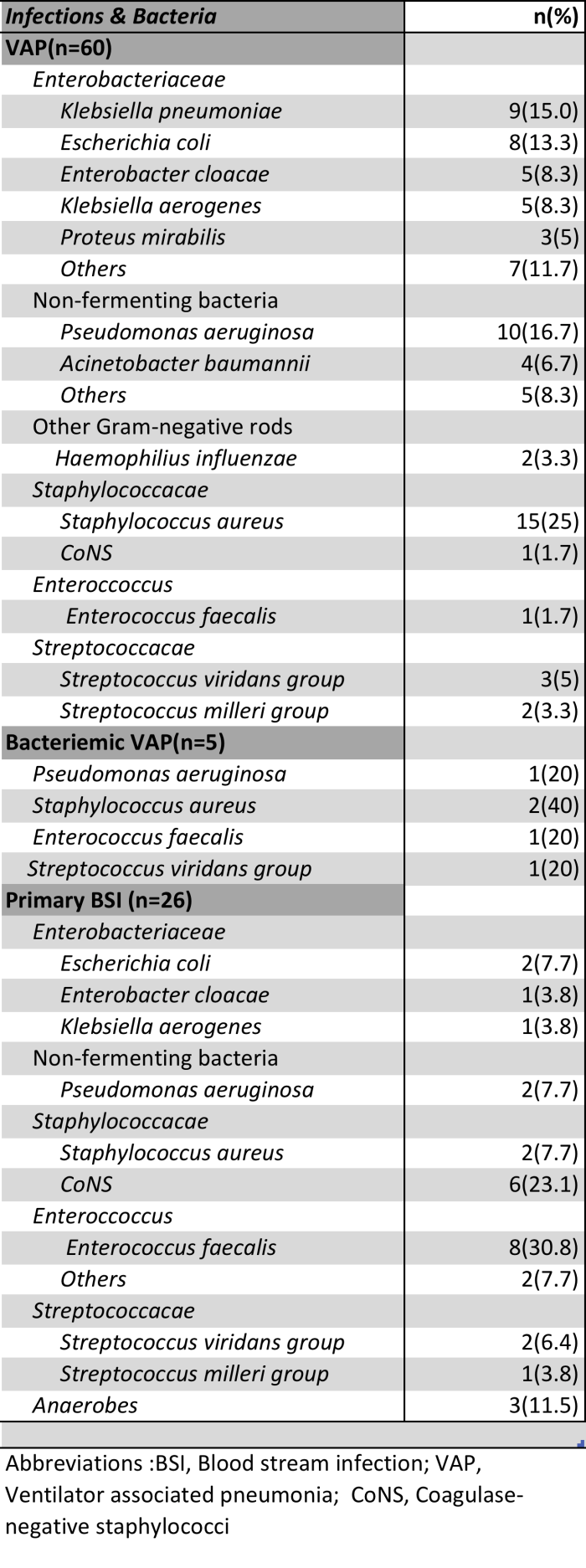

Supplement: S2 Table — (DOCX) [file pone.0250728.s003.docx]
