# Supplementary material for: Two original observations concerning bacterial infections in COVID-19 patients hospitalized in intensive care units during the first wave of the epidemic in France
Source: PLoS One. 2021 Apr 29;16(4):e0250728. doi: 10.1371/journal.pone.0250728 (PMC8084132; doi:10.1371/journal.pone.0250728)

S3 Table. Description of patients and bacteria of patient with or without a persistent ventilator-associated pneumonia (VAP) at day 7 post initial VAP diagnosis.


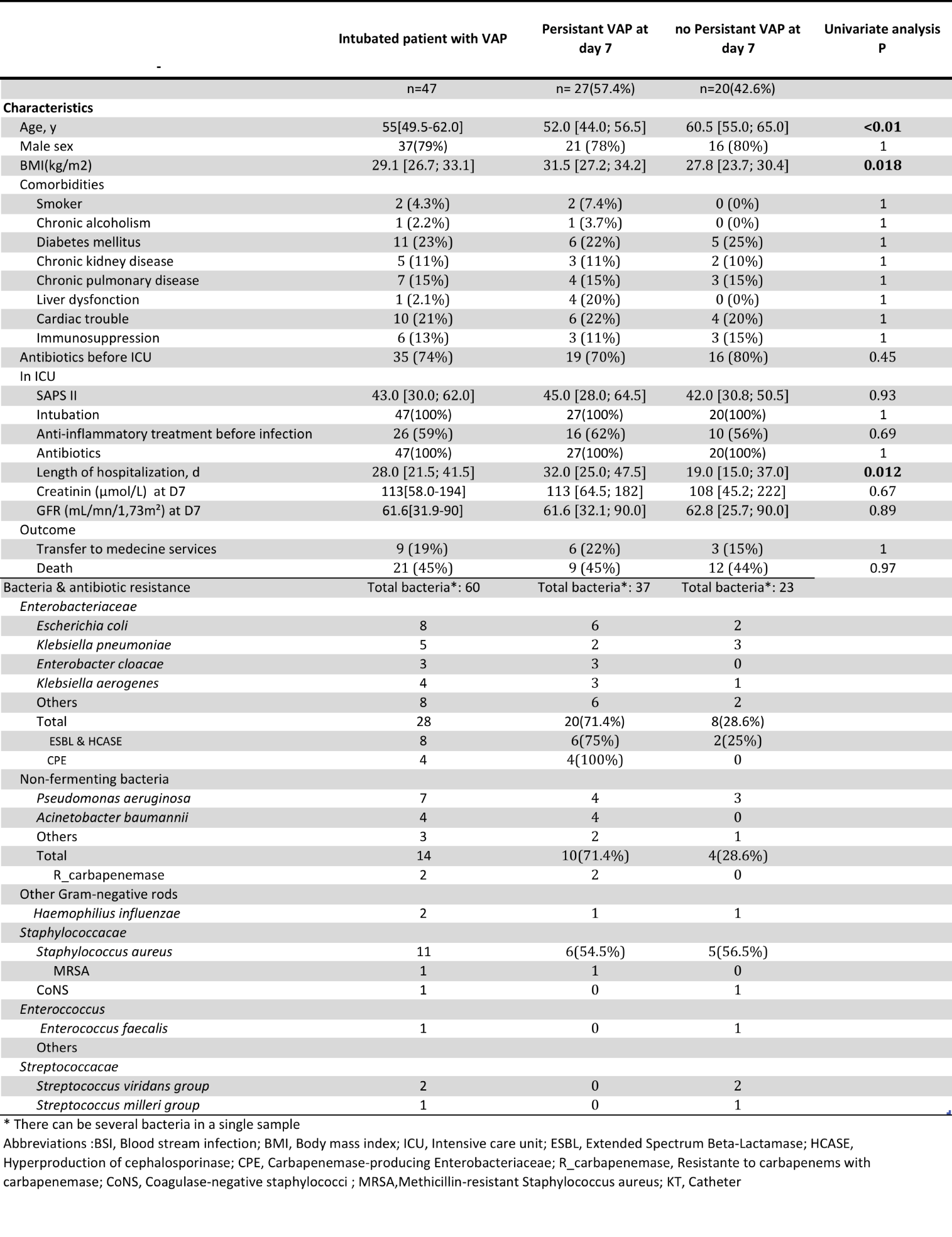

Supplement: S3 Table — (DOCX) [file pone.0250728.s004.docx]
